# Expression of programmed cell death protein 1 and T‐cell immunoglobulin‐ and mucin‐domain‐containing molecule‐3 on peripheral blood CD4+CD8+ double positive T cells in patients with chronic hepatitis C virus infection and in subjects who spontaneously cleared the virus

**DOI:** 10.1111/jvh.13108

**Published:** 2019-04-29

**Authors:** Kamila Caraballo Cortés, Sylwia Osuch, Karol Perlejewski, Agnieszka Pawełczyk, Justyna Kaźmierczak, Maciej Janiak, Joanna Jabłońska, Khalil Nazzal, Anna Stelmaszczyk‐Emmel, Hanna Berak, Iwona Bukowska‐Ośko, Marcin Paciorek, Tomasz Laskus, Marek Radkowski

**Affiliations:** ^1^ Department of Immunopathology of Infectious and Parasitic Diseases Medical University of Warsaw Warsaw Poland; ^2^ Clinic for Infectious, Tropical Diseases and Hepatology Medical University of Warsaw Warsaw Poland; ^3^ Department of Laboratory Diagnostics and Clinical Immunology of Developmental Age Medical University of Warsaw Warsaw Poland; ^4^ Outpatient Clinic Warsaw Hospital for Infectious Diseases Warsaw Poland; ^5^ Department of Infectious Diseases Medical University of Warsaw Warsaw Poland

**Keywords:** DP T cells, hepatitis C virus, PD‐1, Tim‐3

## Abstract

Chronic hepatitis C virus (HCV) infection is characterized by increased proportion of CD4+CD8+ double positive (DP) T cells, but their role in this infection is unclear. In chronic hepatitis C, immune responses to HCV become functionally exhausted, which manifests itself by increased expression of programmed cell death protein 1 (PD‐1) and T‐cell immunoglobulin‐ and mucin‐domain‐containing molecule‐3 (Tim‐3) on T cells. The aim of our study was to determine PD‐1 and Tim‐3 phenotype of DP T cells in subjects with naturally resolved and chronic HCV infection. Peripheral blood mononuclear cells from 16 patients with chronic infection and 14 subjects who cleared HCV in the past were stained with anti‐CD3, anti‐CD4, anti‐CD8, anti‐PD‐1 and anti‐Tim‐3 antibodies and, in 12 HLA‐A*02‐positive subjects, MHC class I pentamer with HCV NS3_1406_ epitope. In chronic and past HCV infection, proportions of total DP T cells and PD‐1+ DP T cells were similar but significantly higher than in healthy controls. DP T cells were more likely to be PD‐1+ than either CD4+ or CD8+ single positive (SP) T cells. HCV‐specific cells were present in higher proportions among DP T cells than among CD8+ SP T cells in both patient groups. Furthermore, while the majority of HCV‐specific DP T cells were PD‐1+, the proportion of HCV‐specific CD8+ T cells which were PD‐1+ was 4.9 and 1.9 times lower (chronic and past infection, respectively). PD‐1 and Tim‐3 were predominantly expressed on CD4^high^CD8^low^ and CD4^low^CD8^high^ cells, respectively, and co‐expression of both markers was uncommon.

**Abbreviation**
DP: double positive
HBV: hepatitis B virus
HCV: hepatitis C virus
HIV: human immunodeficiency virus
LCMV: lymphocytic choriomeningitis virus
RT: room temperature
SP: single positive
PBMC: Peripheral blood mononuclear cells
PD‐1: programmed cell death protein 1
Tim‐3: T‐cell immunoglobulin‐ and mucin‐domain‐containing molecule‐3

## 1 INTRODUCTION

Adaptive immune responses play a critical role in the outcome of hepatitis C virus (HCV) infection.^1^ Strong and HCV‐specific CD8+ and CD4+ cellular immunity is indispensable for the spontaneous clearance of HCV infection, which is observed in 20%‐50% of patients with newly acquired infection but is rare in patients with an already established chronic infection.^2^, ^3^, ^4^, ^5^

Suppression of various mechanisms resulting in suboptimal virus‐specific T‐cell responses has been described for a number of chronic viral infections such as hepatitis B virus (HBV),^6^, ^7^, ^8^ HCV,^9^ lymphocytic choriomeningitis virus (LCMV)^10^ and human immunodeficiency virus (HIV).^11^, ^12^, ^13^ One of the major mechanisms driving the persistence of HCV infection is T‐cell exhaustion which results in weak antigen‐specific T‐cell responses.^14^, ^15^ These defects, which are due to continuous antigen stimulation, progress with the duration of infection and are accompanied by increased expression of inhibitory molecules such as programmed cell death protein 1 (PD‐1) and T‐cell immunoglobulin‐ and mucin‐domain‐containing molecule‐3 (Tim‐3).^15^, ^16^, ^17^

The existence of peripheral blood CD4+ CD8+ double positive (DP) T cells was described both in humans and animals such as rats, mice, chickens, monkeys and swine.^18^, ^19^ Two subpopulations of these cells are distinguished: CD4^high^CD8^low^ and CD4^low^CD8^high^, reflecting the predominance of either CD4 or CD8 expression on their surface.^20^ Although the origin of these cells is still debatable, dominant expression (ie 99%) of CD8αβ heterodimer rather than CD8αα homodimer on their surface implies they are derived from thymic and not gut environment.^20^ DP T cells are mostly functional/effector memory cells specific for antigens of pathogens encountered throughout life.^21^ In healthy blood donors, these cells constitute around 1% of CD3+ cells].^21^ However, increased frequencies of up to 20% were reported in chronic viral infections and these cells were found to represent highly proliferative and active population expressing FasL and IFN‐γ.^22^, ^23^, ^24^, ^25^ Due to the activated phenotype exhibited by DP T cells, they could be hypothetically prone to apoptosis. However, levels of TUNEL expression in DP T cells and CD8^+^ T cells were reported to be similar.^20^ DP T cells (predominantly CD4^high^CD8^low^) are commonly found in peripheral blood and liver in patients with chronic HCV infection.^21^, ^26^ When compared to their single positive (SP) CD4+ or CD8+ counterparts, DP T cells were found to display shorter telomeres indicating they experienced more cell divisions.^21^ In the available animal model of HCV infection (chimpanzee), the proportion of DP T cells negatively correlated with serum viral load, suggesting their involvement in the control of HCV infection.^21^ Information on DP T cells in HCV infection is still limited, however. In particular, the exhaustion phenotype of these cells, their specificity towards HCV antigens and their prevalence in subjects who spontaneously recovered from HCV infection have not been reported.

In our study, we analysed exhaustion markers expression on total and HCV‐specific DP T cells in patients with chronic HCV infection, in subjects who spontaneously cleared HCV infection, and in healthy controls.

## 2 MATERIALS AND METHODS

### 2.1 Patients

Sixteen patients with recently diagnosed chronic HCV infection and 14 subjects who spontaneously cleared HCV infection in the past were recruited among outpatients of the Warsaw Hospital for Infectious Diseases. The latter patients were referred because of anti‐HCV‐positive status but were eventually found to be HCV‐RNA‐negative and without any evidence of liver disease. Eleven healthy anti‐HCV‐negative volunteers with no evidence of liver disease (normal ALT activity levels) served as controls. Peripheral blood mononuclear cells (PBMCs) were isolated from 10 mL of EDTA‐anticoagulated blood by density gradient centrifugation (Lymphoprep, Stemcell Technologies Inc). The presence of HCV‐RNA in plasma was verified by qualitative PCR test (COBAS Amplicor Assay, version 2.0; Roche Molecular Systems, Inc). HCV genotype was determined by Inno‐LiPA HCV II (Innogenetics NV). Viral load was measured by RealTime HCV assay (Abbott) of sensitivity: 12 IU/mL. Some clinical and virological characteristics of patients and controls are presented in Table 1.

**Table 1:** Clinical and virological characteristics of patients and controls

Patients	Sex [male/female]	Median age, yrs (range)	Genotype 1b	Median serum alanine aminotransferase activity [U/L] (range); normal values: 7‐56 U/L	Median viral load [U/mL] (range)
Chronic HCV infection (n = 16)	5/11	52.3 (28‐61)	15[Link]	53.0 (29‐239)	5.85×10^5^ (2.14×10^3^‐3.19×10^6^)
Past spontaneous recovery from HCV infection (n = 14)	4/10	46.6 (26‐75)	N/A	27.0 (10‐48)	N/A
Healthy controls (n = 11)	3/8	49.0 (29‐70)	N/A	24 (12‐51)	N/A

Note

Abbreviation: N/A, not applicable or not available

One patient was infected with genotype 3

### 2.2 Antibodies and pentamers

Surface mouse anti‐human anti‐CD3‐peridinin‐chlorophyll protein‐Cyanine5.5 (PERCP‐CY5.5), anti‐CD4‐BD Horizon V500, anti‐CD8‐fluorescein isothiocyanate (FITC), anti‐PD‐1‐Alexa Fluor® 647 and anti‐Tim‐3‐phycoerythrin (PE) antibodies were purchased from BD Pharmingen. Anti‐CD8‐FITC LT4 clone antibody and custom Pro5^®^ Recombinant MHC class I Pentamer containing HLA‐A*02‐restricted HCV NS3_1406_ immunodominant epitope KLSGLGLNAV (corresponding to genotype 1b), conjugated with PE, were provided by Pro‐Immune. The latter epitope is one of the most immunogenic in chronically HCV‐infected patients and displays cross‐reactivity to other epitope variants.^27^ Furthermore, IgG1 κ ALEXA 647 and IgG1 κ BV421 isotype controls (BD Pharmingen) were used.

### 2.3 HLA‐A*02 typing

The presence of HLA‐A*02 allele was verified by flow cytometry using anti‐HLA‐A*02‐FITC antibody (BD Pharmingen) and by quantitative PCR as described elsewhere.^28^

### 2.4 T‐cell phenotyping

**Figure 1:**
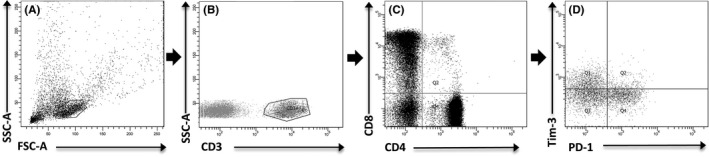
Gating strategy of flow cytometric phenotyping of double positive (DP) (CD4+ CD8+) T cells. A, Dot plot (SSC‐A vs FSC‐A) representing population of acquired PBMC with gated lymphocytes. B, Dot plot (SSC‐A vs Per‐CP) showing CD3+ lymphocytes gated on lymphocytes. C, Dot plot (FITC vs AmCyan) showing population of CD8+ and CD4+ cells gated on CD3+ cells. D, Dot plot (PE vs APC) representing expression of PD‐1 and Tim‐3 on gated DP T cells

Freshly isolated PBMCs (10^6^ cells) were passed through 70 µm cell strainer, resuspended in 100 µL of BD Pharmingen stain buffer with 0.2% (w/v) bovine serum albumin (BD Pharmingen) and pre‐incubated with 10 µL of FcR blocking reagent (Miltenyi Biotec) for 15 minutes at room temperature (RT). Cells from HLA‐A*02‐positive subjects were stained with 10 µL of the pentamer for 10 minutes at RT, washed with 4 mL of MACS buffer (Miltenyi Biotec) and stained with anti‐CD3, anti‐CD4, anti‐CD8, anti‐PD‐1 and anti‐Tim‐3 antibodies for 20 minutes at 4°C. After washing twice with PBS pH 7.2 (Life Technologies), cells were immediately acquired on FACSCanto II instrument (Becton‐Dickinson) and analysed by BD FACSDiva software (Becton‐Dickinson). Cells from HLA‐A*02‐negative subjects were directly stained with antibodies against surface molecules (without pentamer staining) and analysed as above. For data analyses, an initial lymphocyte gate was set based on side scatter (SSC)/forward scatter (FSC) and additional gates (CD3+, CD4+, CD8+, pentamer‐positive, PD‐1+, Tim‐3+ cells) were introduced based on the appropriate fluorescence minus one controls. The gating strategy employed is presented in Figure 1. Results were presented as the percentage of the gated populations.

### 2.5 Statistical analysis

Differences in the proportions of cell subpopulations were analysed by Mann‐Whitney test. All *P*‐values were two‐tailed and considered significant when <0.05.

### 2.6 Ethical statement

The study was approved by the Bioethical Committee of the Medical University of Warsaw (KB/247/2013), and all subjects provided written informed consent.

## 3 RESULTS

### 3.1 CD4+ CD8+ DP T cells

**Figure 2:**
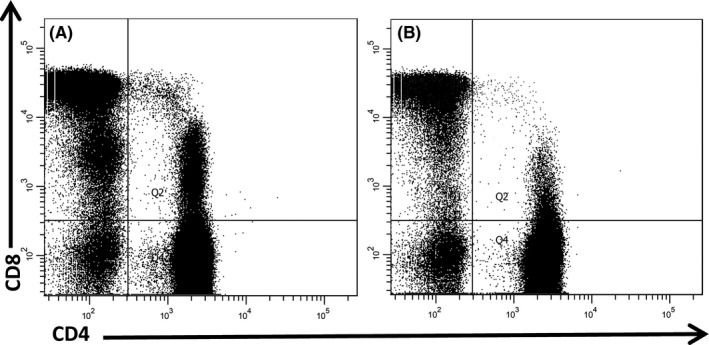
Double positive (DP) (CD4+ CD8+) T cells populations gated on CD3+ cells showing predominant CD4^high^CD8^low^ population in patients #27 (A) and #26 (B). Both patients had chronic HCV infection

The proportion of DP T cells in peripheral blood were similar in patients with chronic infection and in subjects who spontaneously cleared HCV (median 1.9% of CD3+ cells vs 1.8%; NS) but significantly higher than in healthy controls (median 0.7%, *P* < 0.01); (Table 2). Within DP T cells, the population of CD4^high^CD8^low^ cells was 2.7‐fold higher (mean) than the population of CD4^low^CD8^high^ cells in both groups of patients. However, in healthy controls, it was the population of CD4^low^CD8^high^ cells that predominated (Table 2). Examples of dot plots of DP T‐cell populations in two patients with chronic HCV infection are shown in Figure 2.

**Table 2:** Double positive (DP) (CD4+ CD8+) T cells in peripheral blood of 16 patients with chronic HCV infection and in 14 subjects who spontaneously cleared HCV in the past

	DP T cells as percentage of CD3+ cells [mean (median); range]	CD4^high^CD8^low^ DP T cells as percentage of total DP T cells [mean (median); range]	CD4^low^CD8^high^ DP T cells as percentage of total DP T cells [mean (median); range]	Ratio of CD4^high^CD8^low^ to CD4^low^CD8^high^ [mean (median); range]
Chronic HCV infection (n = 16)	2.6 (1.9); 0.7‐6.8	67.5 (73.5); 31.4‐91.0	24.6 (20.7); 5.6‐58.3	2.7 (3.6); 0.5‐16.1
Past HCV infection (n = 14)	2.4 (1.8); 0.7‐11.3	69.0 (65.8); 38.1‐95.5	25.6 (27.1); 2.8‐48.5	2.7 (2.4); 0.8‐34.1
Healthy controls (n = 11)	0.9 (0.7); 0.3‐1.6	33.2 (32.5); 12.3‐53.5	47.2 (50.8); 20.2‐71.9	0.7 (0.6); 0.2‐1.9

### 3.2 PD‐1 and Tim‐3 phenotype of CD4+ CD8+ DP T cells and CD4+/CD8+ SP T cells

**Figure 3:**
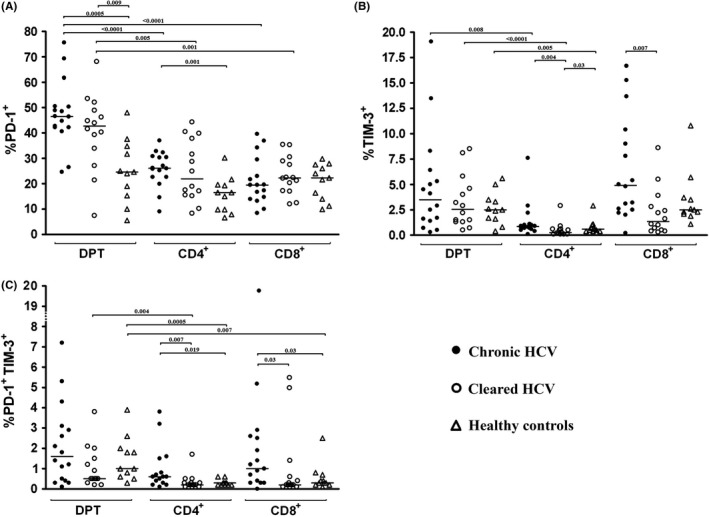
Percentage of double positive (DP) T (CD4+ CD8+) cells expressing PD‐1 (A), Tim‐3 (B) and both PD‐1 and Tim‐3 (C) in peripheral blood of 16 patients with chronic HCV infection, 14 subjects who spontaneously cleared HCV and in 11 healthy controls. Horizontal lines represent median values

The proportion of DP T cells expressing PD‐1 was similar in patients with chronic and past HCV infection (median 46.5% vs 42.7%, NS), but significantly higher than among healthy controls (median 24.6%, *P* < 0.01; Figure 3A). Furthermore, expression of PD‐1 both in chronic and past HCV infection was more common on DP T cells than on CD4+ or CD8+ SP T cells (*P* < 0.01). However, this was not the case in healthy controls.

The proportion of DP T cells expressing Tim‐3 was similar in all groups including healthy controls and higher than the proportion of SP CD4+ cells expressing Tim‐3 (*P* < 0.01; Figure 3B). However, the proportion of both SP CD4+ and SP CD8+ cells expressing Tim‐3 was significantly higher in chronic than in past infection (*P* < 0.01; Figure 3B).

Co‐expression of both PD‐1 and Tim‐3 exhaustion markers on CD4+ and CD8+ SP T cells was significantly more common in chronic infection than either in past infection or in healthy controls (*P* < 0.05; Figure 3C). Furthermore, DP T cells were more likely to be PD‐1+ Tim‐3+ than either SP CD4+ or CD8+ cells, although not all differences were statistically significant (Figure 3C).

### 3.3 PD‐1 and Tim‐3 phenotype of CD4^high^CD8^low^ and CD4^low^CD8^high^ DP T‐cell subpopulations

**Figure 4:**
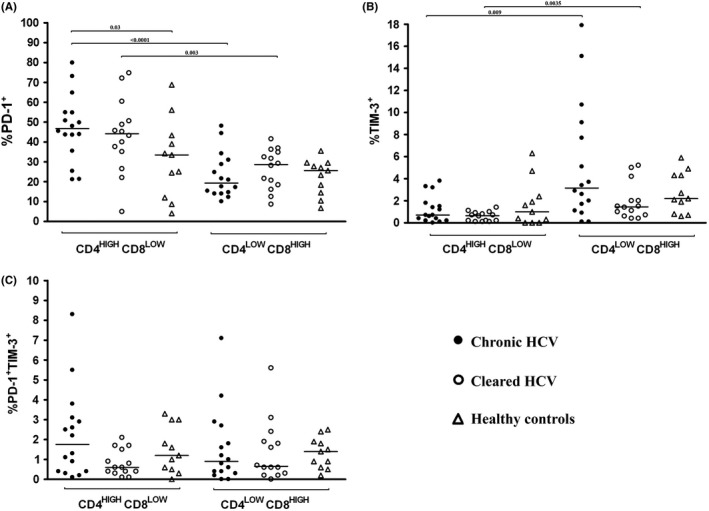
Percentage of CD4^high^CD8^low^ and CD4^low^CD8^high^ double positive (DP) (CD4+ CD8+) T cells expressing PD‐1 (A), Tim‐3 (B) and both PD‐1 and Tim‐3 (C) in 16 patients with chronic HCV infection, 14 subjects who spontaneously cleared HCV and in 11 healthy controls. Horizontal lines represent median values

Programmed cell death protein 1 was present on higher proportion of CD4^high^CD8^low^ DP T cells than CD4^low^CD8^high^ DP T cells both in chronic (median 46.7% vs 19.3%; *P* < 0.0001) and past (median 44.2% vs 28.6%; *P* = 0.003) infection (Figure 4A). Conversely, Tim‐3 was present on higher proportion of CD4^low^CD8^high^ DP T cells compared to CD4^high^CD8^low^ DP T cells, both in chronic (median 3.1% vs 0.7%; *P* = 0.009) and past (median 1.4% vs 0.6%; *P* = 0.0035) infection (Figure 4B). These differences were not present among healthy controls.

There were no significant differences between chronic and past HCV infection in the proportions of PD‐1+ or Tim‐3+ CD4^high^CD8^low^ and CD4^low^CD8^high^ populations. However, PD‐1+ CD4^high^CD8^low^ cells were significantly more prevalent in chronic infection than in healthy controls. Furthermore, there were no significant differences between chronic and past HCV infection in the proportions of PD‐1+ Tim‐3+ CD4^high^CD8^low^ and CD4^low^CD8^high^ populations.

### 3.4 PD‐1 exhaustion phenotype of HCV‐specific DP T cells and CD8+ SP T cells

Hepatitis C virus‐specific cells were analysed in 12 patients with HLA‐A*02 allele: three were chronically infected, while nine spontaneously recovered from HCV infection (Table 3). In chronic infection, 0.2%‐2.5% of DP T cells and 0.006%‐0.098% of CD8+ SP T cells were specific for HCV NS3_1406_ epitope KLSGLGLNAV. The HCV‐specific DP T cells were found to be mainly PD‐1+ (mean 51.3%), while only 10.5% of HCV‐specific CD8+ SP T cells were PD‐1+.

**Table 3:** HCV‐specific cells among double positive (DP) (CD4+ CD8+) and single positive (SP) CD8+ T cells and their PD‐1 expression in three patients with chronic HCV infection and in nine patients who spontaneously cleared HCV

Patient ID	DP T cells specific to HCV[Link][%]	CD8+ SP T cells specific to HCV[Link] [%]	HCV‐specific DP T cells with expression of PD‐1 [%]	HCV‐specific CD8+ SP T cells with expression of PD‐1 [%]
Chronic HCV infection (n = 3)				
20	2.5	0.006	55.6	0
25	0.2	0.098	26.9	7.3
26	1.5	0.082	71.4	24.2
Past HCV infection (n = 9)				
3	0.2	0.133	73.3	12.3
12	0.6	0.073	76.9	39.0
17	0.5	0.015	81.8	60.0
22	0.5	0.012	46.2	22.2
24	0.2	0.044	16.7	18.4
19	1.8	0.025	39.0	13.3
15	1.5	0.024	55.3	23.1
29	0.4	0.163	68.2	25.1
32	0.7	0.003	36.4	40.0

NS3_1406_ epitope KLSGLGLNAV

In subjects who recovered from HCV infection, 0.17%‐1.8% of DP T cells and 0.003%‐0.163% of CD8+ SP T cells were reactive to HCV NS3_1406_ epitope. Again, the HCV‐specific DP T cells were found to be mostly PD‐1+ cells (mean 54.9%) which was in contrast to HCV‐specific CD8+ SP cells, of which only 28.2% were PD‐1+.

**Figure 5:**
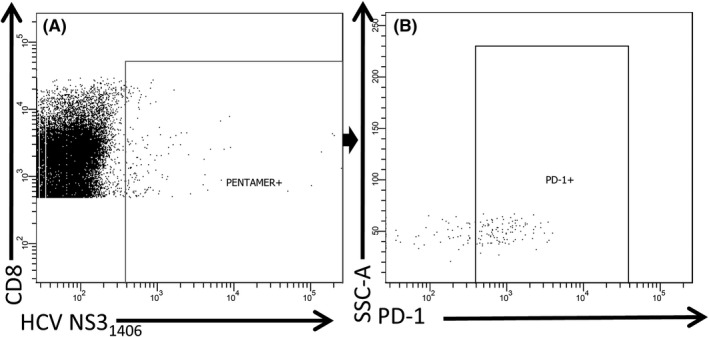
Population of double positive (DP) (CD4+ CD8+) T cells specific for HLA‐A*02‐restricted HCV NS3_1406_ KLSGLGLNAV epitope (A) together with PD‐1 expression (B) in patient #29 who cleared HCV infection in the past

An analysis of DP T‐cell population specific for HLA‐A*02‐restricted HCV NS3_1406_ KLSGLGLNAV epitope together with PD‐1 expression in patient #29 is shown in Figure 5.

## 4 DISCUSSION

In the current study, we characterized the population of CD4+ and CD8+ DP T cells according to their PD‐1 and Tim‐3 expression phenotype and specificity to HCV NS3 epitope in patients with chronic HCV infection and in patients who spontaneously cleared the infection. We also analysed a group of healthy control subjects with no evidence of HCV infection or liver disease.

We found the proportion of DP T cells in chronic and past infection to be similar (1.9% vs 1.8%) and these numbers are close to those reported by Nascimbeni et al^21^ who found the frequency of DP T cells in HCV‐infected patients to be 1.2%. We also found a clear predominance of CD4^high^CD8^low^ over CD4^low^CD8^high^ subset both in chronic and past infection which is also consistent with other studies on HCV infection.^21^, ^26^ Such similar distribution of DP T cells and their subsets in chronic and past infection suggests their long‐term preservation after exposure to HCV. Indeed, it has been shown that DP T cells frequently have central memory phenotype as demonstrated by the expression of CCR7 and CD45RO.^21^ Markedly lower proportion of these cells (0.7%) and reverse proportion of CD4^high^CD8^low^ subsets found in healthy controls imply that the observed effects were indeed due to HCV exposure. The proportions of DP T cells expressing PD‐1 and/or Tim‐3 were found to be similar in chronic and past HCV infection which suggests that expression of these markers on these cells is long‐lasting and perhaps irreversible. Since healthy controls had significantly lower PD‐1 expression, PD‐1 expression on DP T cells seems to be highly affected by exposure to HCV.

There are no published data regarding the PD‐1 and Tim‐3 phenotype of DP T cells but a number of studies analysed SP T cells. Thus, studies on chronic HIV infection suggest that PD‐1 is a marker of early T‐cell exhaustion, representing a stage of already impaired proliferation, but still relatively well‐preserved T‐cell function, including cytokine production. 29 In contrast, Tim‐3 seems to be a marker of more advanced T‐cell exhaustion associated with HIV disease progression^30^ and was also shown to be up‐regulated in HCV infection.^31^

In our study, the proportions of CD8+ SP T cells expressing Tim‐3 were higher in chronic than in past infection, which suggests that the expression of Tim‐3 on these cells is maintained for the duration of infection. Indeed, once the infection is cleared, it may be possible to achieve physiological expression levels as evidenced by similar proportions of Tim‐3+ CD8+ SP T cells in past infection and in healthy controls. Similar results were reported by McMahan et al^9^ who found patients with chronic HCV infection to have higher frequency of Tim‐3‐expressing CD8+ SP T cells than patients who cleared the infection.

We found relatively low percentage of PD‐1+ Tim‐3+ T cells across all analysed groups (Figure 3) which is compatible with the existence of two distinct cell subpopulations expressing either Tim‐3 or PD‐1. Similar findings were reported by Jones et al in HIV infection.^30^ Co‐expression of inhibitory molecules PD‐1 and Tim‐3 may characterize a set of particularly exhausted T cells, and it has been shown that their presence is associated with poor virological outcome of acute HCV infection in humans.^9^ Similarly, a recent study using murine LCMV animal model demonstrated that dual expression of these inhibitory receptors is associated with progression to chronicity.^10^ In line with these findings, we found significantly higher proportions of PD‐1+ Tim‐3+ CD4+ and PD‐1+ Tim‐3+ CD8+ SP T cells in chronic HCV infection than in past infection or healthy controls.

Interestingly, we found that CD4^high^CD8^low^ and CD4^low^CD8^high^ subsets differed with respect to exhaustion markers expression, both in chronic and past HCV infection. While almost half of CD4^high^CD8^low^ cells were PD‐1+, only about 20% of CD4^low^CD8^high^ cells expressed this marker. Conversely, CD4^low^CD8^high^ cells were more prone to express Tim‐3 than CD4^high^CD8^low^ cells (3.1% vs 0.7% in chronic infection and 1.4% vs 0.6% in past infection). Importantly, this was not the case in healthy controls, which suggests that the observed effects were due to HCV exposure.

Expression of Tim‐3 was higher on CD8+ SP T cells than on CD4+ SP T cells in all analysed groups, which suggests that CD4^low^CD8^high^ and CD4^high^CD8^low^ subsets might have evolved from CD8+ and CD4+ SP T cells, respectively, and thus may display similar exhaustion markers expression as the cells of origin. This speculation is supported by studies showing that thymus‐derived SP cells up‐regulate either CD4 or CD8 upon activation in HIV infection.^32^, ^33^ Similarly, at least one study traced low expression of CD4 in vitro to CD8 T‐cell origin.^20^

Analysis of T‐cell populations specific for HLA‐restricted HCV NS3_1406_ epitope KLSGLGLNAV revealed that HCV‐reactive cells were of higher frequency among DP T cells. This suggests that DP T cells play an active and perhaps critical role in antiviral response. This phenomenon is clearly not HCV‐specific as similar findings were described by Zloza et al who investigated the functionality of human DP T in response to CMV peptide (pp65) priming and found that the percentage of DP T cells recognizing CMV pp65 tetramer was ∼19‐fold higher than of CD8+ SP T cells.^20^

Similar proportions of HCV‐specific DP T cells in chronic and past HCV infection found in our study suggest that once generated by antigen stimulation, these cells are maintained regardless of the infection outcome. However, higher expression of PD‐1 on HCV‐specific DP T cells, when compared to HCV‐specific CD8+ SP T cells, suggests that the likely strong antiviral functions of the former cells may come at a cost of incurring an inhibitory phenotype.

## 5 CONCLUSIONS

In summary, we found total DP T‐cell proportions and PD‐1+ DP T‐cell subpopulations to be similar in chronic and past HCV infection but higher than in healthy controls. Furthermore, the expression of PD‐1 on total DP T cells was higher than on either CD4+ or CD8+ SP T cells. HCV‐specific cells were of higher frequency among DP T cells and were more likely to express PD‐1 when compared to HCV‐specific CD8+ SP T cells. Our findings suggest that DP T cells play an important, perhaps critical role in the immune response to HCV, but at the same time seem to be prone to incur an inhibitory phenotype.

## CONFLICT OF INTERESTS

None declared.
